# A systematic review of the economic evidence surrounding the management of alcohol withdrawal

**DOI:** 10.1111/dar.14053

**Published:** 2025-04-14

**Authors:** Darren Quelch, Rachel Granger, Huw Lloyd‐Williams, Arlene Copland, Gareth Roderique‐Davies, Bev John, Rhiannon Tudor Edwards

**Affiliations:** ^1^ Addictions Research Group, Applied Psychology Research and Innovation Group Faculty of Life Sciences and Education, University of South Wales Pontypridd UK; ^2^ Alcohol Care and Clinical Toxicology Team Sandwell and West‐Birmingham Hospital NHS Trust, City Hospital Birmingham UK; ^3^ Centre for Health Economics and Medicines Evaluation Bangor University Bangor UK; ^4^ Wavehill Social and Economic Research Ltd. Aberaeron UK

**Keywords:** alcohol withdrawal, alcohol withdrawal management, cost‐effectiveness, economics, systematic review

## Abstract

**Issues:**

Alcohol withdrawal syndrome (AWS) is a medical emergency associated with lengthy hospital stays and an increased frequency of alcohol‐related hospital admissions. Rising numbers of alcohol‐related health presentations and limited resources of alcohol treatment services necessitate the implementation of both cost‐effective and clinically effective interventions.

**Approach:**

A systematic literature search was conducted to review the economic evidence base for AWS interventions. A search of PubMed, Medline, Embase, Web‐of‐Science and Proquest identified 6347 articles. Following duplicate removal, 5250 English language papers were screened; 58 papers met eligibility criteria. Fifty papers were excluded at full‐text screening; 8 papers were included. A novel logic model describing factors impacting clinical and cost‐effectiveness of AWS management was developed.

**Key Findings:**

The United States (3), the United Kingdom (3), France (1) and Switzerland (1) based studies took primarily a health sector perspective, with most reporting on cost savings, rather than full health economic evaluations. Both patient‐ or symptom‐specific guidelines and outpatient treatment reduce service costs in select patient populations, without impacting on treatment outcomes. Additional psychological outpatient support may also be a cost‐effective addition to treatment.

**Implications:**

Where clinically suitable, early transition of AWS treatment to outpatient settings, alongside implementation of patient‐ or symptom‐specific treatment guidelines, both may improve the cost‐effectiveness of alcohol treatment services. Significant heterogeneity among current study methodology, patient population and poor‐quality economic evidence means further studies are required.

**Conclusion:**

To develop a more robust understanding of cost and clinical‐effectiveness, we propose a transdisciplinary research agenda between health economics, academic expertise and AWS services to address the current evidence gap in this area.

## 1 INTRODUCTION

In 2019–2020, 608,416 people were living with alcohol dependence in England [1]; of those engaged in treatment, 13,349 people received pharmacological interventions, mainly to facilitate withdrawal from alcohol [2]. Alcohol withdrawal is a medical emergency associated with lengthy hospital stays and an increased frequency of alcohol‐related hospital admissions. Alcohol withdrawal syndromes (AWS) also carry an increased risk of medical complications such as alcohol withdrawal seizures and delirium tremens, and mortality [1–3]. Inpatient AWS management typically involves: (i) comprehensive assessment by an individual with expertise in the management of alcohol‐related health problems; (ii) provision of thiamine; (iii) symptom management, often through the administration of benzodiazepines; and (iv) psychological support [3].

Planned or elective alcohol detoxification may be performed through community or third‐sector services, acute hospital services, residential rehabilitation settings, ambulatory or home settings. The setting for withdrawal management is often determined by clinical risk. An abrupt reduction in alcohol intake, due to medical comorbidity or lack of access, for example, often leads to the development of severe physical withdrawal symptoms, necessitating acute medical service involvement. Furthermore, a history of alcohol withdrawal‐related seizures or delirium tremens may also increase the need for medical oversight during the withdrawal process. However, where sufficient psycho‐social support is in place, and the risk of severe withdrawal syndromes is low, ambulatory or home detoxification is considered a safe and effective alternative to inpatient detoxification [4]. The efficacy of acute, residential inpatient and outpatient withdrawal management has been reviewed. Direct comparison of long‐term outcomes between inpatient and home detoxification is not possible, secondary to the different populations' medical and psychosocial characteristics. However, Stockwell et al. [4] reported a reduction in medication demand and comparable levels of drop‐out from rehabilitation and complication rates in home detoxification. Home detoxification may also lead to a reduction in perceived stigma and maintained engagement, and be more convenient to patients and various services, while sparing acute service resources through utilisation of existing support networks [5].

Alcohol is widely considered to represent the most harmful substance of misuse at a societal level [6]. The financial implications of alcohol misuse have recently been reviewed. In England alone, alcohol costs society £27.44 billion, of which £4.91 billion are National Health Service and health‐care associated costs, £14.58 billion are crime and disorder related, £5.1 billion are attributable to absenteeism, presenteeism and unemployment, and a further £2.9 billion are associated with alcohol‐related costs to social services [7]. Globally, costs relating to both direct and indirect harms associated with alcohol equate to 2.6% of gross domestic product [9].

The number of alcohol‐related presentations to emergency departments is increasing [10]. In 2022–2023, 1.13% of total attendances to emergency departments in England were related to a primary or secondary diagnosis code associated with alcohol excess or dependence (total = 117,525 presentations due to a diagnosis of acute alcohol intoxication, alcohol dependence, toxic effects of alcohol, alcohol withdrawal‐induced convulsions or uncomplicated alcohol withdrawal) [11]. Australia and New Zealand report that alcohol‐related health conditions constitute 13% and 16% of total presentations to emergency departments, respectively [12]. The proportion of those developing severe alcohol withdrawal among these groups may range between 2% and 7%, reaching as high as 20% in specialist treatment centres [13]. As such, health‐care resource use and costs related to the management of alcohol withdrawal, both in acute and non‐acute settings, are likely to be significant. However, to our knowledge, a systematic review of published studies detailing the financial costs associated with delivering alcohol withdrawal services has not yet been performed.

In alignment with recommendations from the National Institute for Health and Care Excellence relating to both the development of clinical guidance [8] and determination of value for money for health‐care interventions [14], we aim to review the evidence base surrounding the financial implications of delivering structured alcohol withdrawal management interventions through the conduct of a systematic literature search. Interventions across acute, residential, community and home‐withdrawal settings were included. Studies where costs associated with the attainment of long‐term abstinence and sobriety were implicated in the economic evaluation of a service were excluded.

## 2 METHODS

### 2.1 Pre‐registration

A protocol for this systematic review was registered on Prospero on 26 September 2023: Darren Quelch, Rachel Granger, Huw Lloyd‐Williams, Gareth Roderique‐Davies, Bev John, Rhiannon Tudor Edwards. Interventions for the management of alcohol withdrawal syndromes: A systematic review of economic evaluation studies. PROSPERO 2023 CRD42023465992. Available from: https://www.crd.york.ac.uk/prospero/display_record.php?ID=CRD42023465992.

### 2.2 Inclusion and exclusion criteria

#### 2.2.1 Study type and setting

Clinical audits, quality improvement and service evaluation studies with an economic evaluation, cost‐effectiveness and social return on investment perspective (reporting outcomes such as financial benefit, cost‐benefit ratio, quality‐adjusted life years, length of stay, health‐care professional hours, intervention costs, e.g.) were included. Although systematic and narrative reviews were excluded, reference lists of any relevant reviews were scanned to identify additional citations. Studies performed across a range of service settings were included (Table S1, Supporting Information); preclinical, perinatal and paediatric service reports were excluded.

#### 2.2.2 Patients and interventions

Individuals over the age of 18 years with an alcohol‐related diagnosis suggestive of dependence were included. Alcohol dependence was defined as a disorder of regulation of alcohol use arising from repeated or continuous use of alcohol with: (i) features of impaired control over alcohol use; (ii) an increasing precedence of alcohol use over other aspects of life; and (iii) features of tolerance, withdrawal or use of other substances to prevent withdrawal symptoms [15]. Those reporting outcomes from individuals identified as consuming alcohol socially, ‘hazardously’ or recreationally were excluded, as were those focussed on the management of intoxication alone. In order to ensure a broad representation of alcohol withdrawal services and interventions, interventions with a primary focus on the management of alcohol withdrawal were included, for example, medication detoxification regimens, the addition of psychological interventions and complex interventions (those employing multiple treatment modalities). Outcomes from services whose primary aim was consumption reduction, abstinence or sobriety were included unless there was a lack of data reporting surrounding alcohol withdrawal management (e.g., treatment setting, length of stay, number of health‐care professional interactions, number of intervention administrations).

### 2.3 Searches

A systematic review of the published literature was performed across the following platforms: ProQuest—APA PsychInfo, MEDLINE and PubMed Central, Web of Science and Embase. Pre‐defined search terms for either population, intervention, comparator or outcome (PICO) domains were generated ([16]; Table S2). Where appropriate, search engine specific Medical Subject Heading, Emtree and APA Thesaurus of Psychological Index Terms were also used. Searches were combined using the Boolean operators ‘OR’ and ‘AND’. Searches were performed on 6 October 2023. No date restrictions were applied. English and Welsh language‐only limits were applied. Duplicates were detected and removed via bibliographic software [17].

### 2.4 Study selection

**FIGURE 1:**
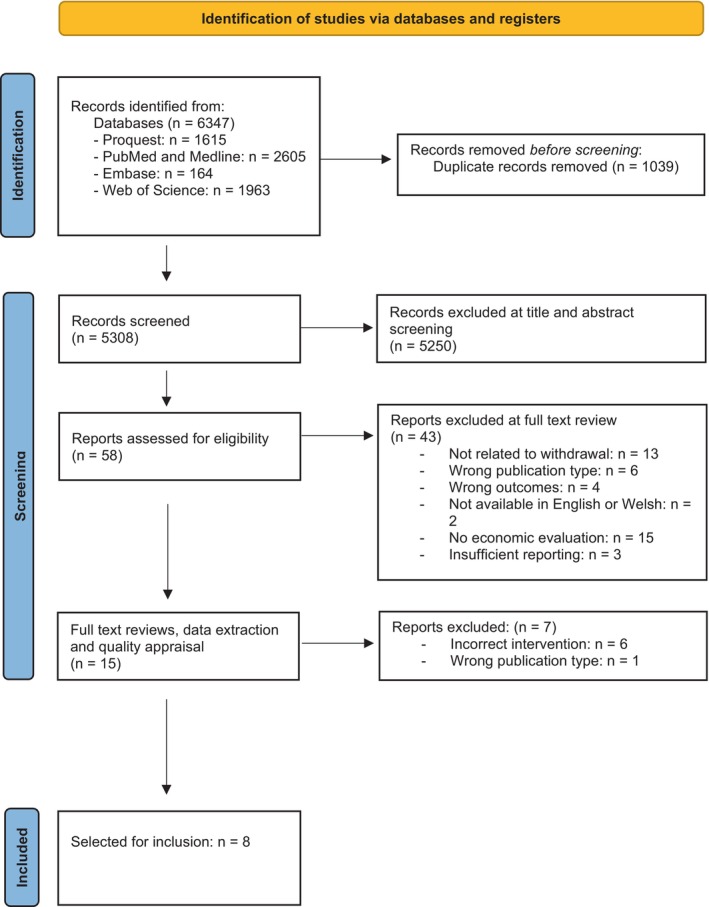
PRISMA study selected flowchart [19]. *Source:* Page et al. [44].

A further round of duplicate removal and initial title and abstract screening against inclusion and exclusion criteria was performed using Rayyan [18]. Paired combinations of authors 1, 2 and 3 performed individual study screening and independent article selection. Where disagreement arose between the first and second reviewers, the third reviewer was consulted to make a consensus decision. Those that met the inclusion criteria from title and abstract screening underwent full‐text review and data extraction; this was performed independently by authors 1 and 2 and reviewed by author 3. Study selection was agreed by consensus by the authorship team. See Figure 1 for study selection flow. Data extraction was performed across the following study variables: publication details, study design and intervention, participant information including sociodemographic variables and alcohol use history, economic evaluation details, alcohol‐related study outcomes and economic evaluation outcomes.

### 2.5 Study outcome management

Clinical, economic and health economic outcomes were grouped by intervention type. Due to the heterogeneity of intervention type, economic evaluation methodology and date of studies, a ratio of the cost of the intervention: cost of TAU was used to enable comparison of economic outcomes. Due to the number of studies included and the limited number of grouped comparisons possible, TAU standardisation was not performed; outcomes (and ratio of the cost of the intervention: cost of TAU) are discussed on a case‐by‐case basis.

### 2.6 Quality appraisal

The Consolidated Health Economic Evaluation Reporting Standards (CHEERS [20]) tool was used to quality assess the studies selected.

## 3 RESULTS

### 3.1 Studies

Six thousand three hundred and forty‐seven studies were retrieved from the systematic searches performed across PubMed and Medline, Embase, Web of Science and Proquest (Figure 1). Following duplicate removal, title and abstract screening, and eligibility review, 15 papers met the inclusion criteria. At the full‐text review, data extraction and quality appraisal stage a further seven were excluded. Eight studies were included in the final data synthesis.

Three studies were performed in the United Kingdom, three in the United States, one study was performed in France and one study in Switzerland. The studies were published between 1983 and 2017. Two studies were performed within acute medical settings [21, 22]; the remaining six were conducted either in a dedicated alcohol treatment setting (including home detoxification performed by community services) or a psychiatric service: namely, a community alcohol service [23], an inpatient detoxification service with outpatient capacity [24], a residential treatment service [25, 26] and an emergency psychiatric outpatient clinic [27]. A single study reported outcomes from a private health‐care setting [28]. Three studies were retrospective in their design; the remainder were prospective: two of which reported results from randomised controlled trials (Table 1).

**TABLE 1:** Summary of included studies.

Author and year (reference)	Design	Setting and population (*n*)	Description of dependence	Intervention (comparator)	Intervention outcome	Currency (year of costing), data source and economic analysis method	Economic outcomes
J Alwyn 2004	Pragmatic randomised trial	Community alcohol team patients (91)	All had SADQ ≥30 81 = 27.4 units/day 10 = 27 units/day but with mean 39 abstinent days/90 days	Psychological intervention over 5–8 day period = motivational interviewing, coping skills training and social support (treatment as usual = five home visits over 5–8 day period for administration of medication only)	Increase in abstinence or >3 drinks per drinking episode compared to ATU at 3 months (58%:24%) and 12 months (39%:9%). Increased mean number of days until first drink compared to TAU (114 days: 52 days). Reduction of total number of units consumed. NNT of 3.13 reported.	GBP (2001–2004) Local NHS trust data (2004) used for the intervention. UK national tariffs (2001) used for control, service cost comparison of intervention with TAU. Costing based on the number of inpatient days, outpatient days and home visits.	Additional costs for intervention of £175 per participant, based on hourly rates for Community Psychiatric Nurse staff costs. No costs were included for staff training. Same contact time for inpatient (14 days) outpatient (5 attendances) and home‐based visits (5 visits) was assumed for both intervention and TAU costs. Costs were calculated using both on local NHS health board and national tariffs: no discussion on cost differences was provided. No costs calculated from NNT improvement.
M Hayashida 1989	Randomised prospective trial	Inpatient and outpatient alcohol detoxification (164)	MAST >5; SADQ >31. Inpatients = 13 years of heavy drinking; outpatients = 14.1 years of heavy drinking	Inpatient = daily adjusted dose BZP, thiamine, group counselling, Alcoholics Anonymous meetings, social therapy PLUS 6 day extension for stabilisation following detoxification. (Outpatient = daily adjusted dose BZP given to be taken as needed, thiamine, self‐reports of consumption and BAC measurements, brief social and withdrawal related counselling).	Significantly more people completed detoxification if were inpatient; treatment duration significantly longer in outpatient group; no difference in seizure frequency significantly, more people remained abstinent in the inpatient group at 1 month; no difference in abstinence rates at 6 months	USD (1988) Service cost data provided by the trial centre and included inpatient medical stay, outpatient appointments, screening visit costs and explicit detoxification charges. Direct costs to participants were collected from surveys. Service costs (including overheads) and marginal costs per treatment of additional patient were calculated. Sensitivity analysis for both program and marginal costs included average costs of outpatient visits only (low) or average cost of outpatient visits, in addition to indirect service costs, patient transport costs and patient opportunity costs (cost of participant's time).	Average service costs for Inpatient were 12‐fold higher than outpatient costs ($3319–3665: $175–388), which was linked to treatment duration (9.2 days: 6.5 days) and corresponding greater residential administration costs and patient opportunity costs. Marginal costs were also significantly higher at 4‐ to 7‐fold for inpatients than outpatients ($756–3047: $190–414). Higher increases of marginal costs for outpatients was due to the increased cost of professional time required for individual home visits, rather than hospital‐based staff for inpatient.
L Hoey 1994	Prospective 6 month observational study^a^	Inpatient medical centre; intensive care unit (50)	Limited – requirement of management of alcohol withdrawal symptoms	Impact of implementation of chlordiazepoxide symptom management guideline (lorazepam intravenous administration versus guideline‐based lorazepam and chlordiazepoxide oral or intravenous decision tree prescribing; Pre‐guideline implementation comparator group previously published elsewhere^a^)	Increased number of patients receiving BZP following implementation of guideline; significant reduction in the number of ICU days	USD (1994) Benzodiazepine treatment (acquisition) costs, based on daily dose. Source of acquisition costs are not provided in the paper.	20‐fold reduction of drug acquisition costs for treatment. Prior to implementation, mean cost BZP therapy per person was $1008.72 (±$1554.45). Following implementation, mean acquisition cost of BZP therapy per person was $59.79 (±$122.92). A reduction of average ICU stay is also recorded (4.1 days: 1.1 days) but associated costs are not calculated.
L Soravia 2017	Retrospective data analysis	Residential alcohol withdrawal treatment centre (301)	AWS arm = Duration of dependence 13.94 years; 109.56 drinks per week; equivalent to 191.73 units/week. TAU arm = duration of dependence 13.12 years; 106.25 drinks per week; equivalent to 185.9 units/week Alcoholics Anonymous	AWS = abridged CIWA‐Ar and symptom‐triggered BZP administration (treatment as usual = fixed regimen of BZP administration)	AWS = reduction in proportion of patients needing medications; acute detoxification duration reduced; reduction in LOS on detoxification unit; duration of BZP administration reduced overall; reduction in dose for adjunct medications	CHF (not stated) Service cost, based on number of days in the detoxification unit, and average rates for 1 day in an acute psychiatric institution in Switzerland of 655.1 ± 106.5 CHF (costing source not stated).	2‐fold reduction in costs was reported due to AWS, in the acute phase (CHF3710.5 ± 1621.7: CHF1797.8 ± 1137.4) and 1.4‐fold reduction for entire stay (CHF12,509.8 ± 4048.1: CHF9059.7 ± 3660.8). Sixty‐six percent reduction in drug usage due to intervention (14.8 mg: 5.1 mg), but not costed for. Staff training and increased monitoring was noted in paper, but not costed.
B Nalpas 2003	Retrospective, non‐randomised descriptive study	Specialist alcohol treatment centres (267)	Limited—number of drinks per day in 3 months preceding withdrawal 19–32; equivalent to 33.3–56.0 units/day	Limited—inpatient admission, medical (including medications and investigations) and non‐medical interventions (no comparator)	Alcohol relapse rate and mean time without relapse differed by treatment centre	EUR (2020) Costs were derived from official refund prices from the French national social security service, calculated in Francs then converted into Euros. Service costs, based on standard average daily rate of €62.20 for hospital ward and employee costs, with mean costs for medical visits, inpatient program, medication costs and investigation costs, provided per centre. Efficiency costs were calculated based on total program care costs divided by mean time in months without relapse per centre.	Costs for residential stay used standard national tariff. Acute treatment costs ranged from €1326 to €1917 (*p* = 0.001), driven by LOS (11–28) days, Total cost ranged from €1832 to €3031, +again driven by level of inpatient stay. There was a wide range of costs per centre reported for inpatient program and supplementary medical visits (€81–259), investigations (€43–347) and drugs (€30–116). Mean time before relapse varied from 3.73–4.94 months. Cost‐effectiveness was approximately €500/month in the third and €658 in the fourth centre.
M Klijnsma 1995	Uncontrolled follow‐up study	Psychiatric emergency clinic (28)	Limited—30% had SADQ score >30; Alcohol Problem Inventory Score 5.25; no history of withdrawal seizures or delirium tremens	Outpatient attendance at a 5‐day program = daily reducing dose of diazepam, structured counselling, mean corpuscular volume and gamma‐glutamyl transferase activity measured (no comparator)	17 had good or improved outcomes following engagement; association between outcome and engagement with voluntary services	GBP (1994) Service costs comparison. Outpatient service costs were calculated based on average study clinic costs for the out‐patient program (12%), with calculated cost for 1 day of attendance used to calculate total outpatient costs. IP service costs were calculated based on inpatient bed daily cost on a general adult psychiatric ward (£117) and average LOS of 12 days.	Cost for outpatient detoxification is estimated to be six‐fold cheaper than estimated inpatient costs (£235:£1404). OP costs were calculated as £47 per day for the clinic (including nursing, medical and secretarial personnel, as well as capital charges), multiplied by 5 for the duration of treatment. IP cost is an estimation, as data was not collected as part of this study and source of data is not stated.
R Longabaugh 1983	Randomised controlled trial	Private psychiatric hospital (260)	Limited—100% had diagnosis of ‘alcoholism’; mean alcoholism index score = 16–17; mean number of years heavily drinking = 15–17; 56%–62% had previously had alcohol related presentations	EIT = completion of detoxification + extended inpatient stay THEN 8 h per week of therapy +2 h of social skills + one film + one lecture per week delivered while continuing to receive treatment as an inpatient (PHT = completion of detoxification + discharge THEN 8 hours per week of therapy +2 h of social skills + one film + one lecture per week delivered while commuting from normal residence)	EIT = Increased abstinent patient number, increase abstinent days, reduction in arrests; 25% had an alcohol related rehospitalisation; Duration of detox the same between treatment and the total number of days for each treatment similar—difference driven by number of attendances to detox clinic = 10.5 in EIT group, 14.6 in PHT; increased engagement in Alcoholics Anonymous	USD (1980) Service costs, calculated from hospital bills of participating patients and included inpatient detox costs, residential room and board, and treatment program attendance. Cost data was also verified by insurance company data.	Average daily charge for the detoxification was $145 for IP and $95 for outpatient services. Initial inpatient detox was comparable for both groups (average $1034). Outpatient program was 1.6‐fold cheaper for EIT for the total program (*p* < 0.001) ($4359: $2700), due to residential costs. PHT had treatment cost ($1000:$1389) due to attending a higher average number of sessions (14.6:10.5). No differences were reported in re‐hospitalisation or multiple readmission outcomes at 6 months from the two arms, although costs for readmission were higher for EIT ($2116:$1689), again driven by residential costs.
J Murdoch 2014	Retrospective evaluation—pre and post‐implementation	Acute hospital trust (50)	Limited—Patients requiring treatment for alcohol withdrawal	Implementation of a symptom‐triggered chlordiazepoxide detoxification protocol (no formal comparator, before and after implementation data; pre‐implementation = non‐symptom triggered protocol)	Statistically significant reduction in LOS, duration of treatment and amount of medication prescribed; reduction in reporting of severe alcohol withdrawal signs (seizures and delirium tremens)	GBP (not specified) Service costs, calculated from average inpatient stay and treatment costs of £300 per day (data source not specified), multiplied by average LOS pre‐ and post‐ implementation of protocol. Hospital billing data was used to calculate the average number of days to complete detox after starting treatment.	Reduction of 2.6‐fold in costs (£1908 pp: £744 pp) due to a 60% reduction of average IP duration for intervention versus TAU (6.36 days: 2.48 days). A 66% change in chlordiazepoxide dose was recorded (563 mg: 167 mg) but change in costs were not calculated.

*Note*: USA standard drink = 14 g of pure ethanol; 1 UK unit = 8 g of pure ethanol.

Abbreviations: AAWS, alcohol withdrawal scale; BAC, blood alcohol concentration; BZP, benzodiazepine; CHF, Swiss Francs; CIWA‐Ar, Clinical Institute Withdrawal Assessment of Alcohol Scale, Revised; EIT, extended inpatient treatment; EUR, Euros (€); GBP, Great Britain Pounds (£); ICU, intensive care unit; LOS, length of stay; MAST, Michigan Alcohol Screening Test; NHS, National Health Service; NNT, number needed to treat; PHT, partial hospital treatment; SADQ, Severity of Alcohol Dependence Questionnaire; TAU, treatment as usual; USD, US dollars ($).

^a^ Comparator group pre‐guideline data published in [20].

### 3.2 Population

Outcomes are reported from 1212 participants across the eight studies. Sex was not reported in three studies; in the remaining five studies, males were disproportionately represented (percentage male population range 56–76%). The mean age of those included in the study was 44.2 years (standard deviation = 2.40 years). Ethnicity, socio‐economic, employment and housing status were not consistently reported.

Reporting surrounding participant alcohol use history and severity of dependence was variable. Dependence on alcohol by the study participants was either confirmed by psychiatric history, objective tools or the clinical requirement for alcohol withdrawal management. The Severity of Alcohol Dependency Questionnaire was used by three studies; all of which reported scores ≥30 suggestive of severe dependence. The Michigan Alcohol Screening Test was used in a single study, reporting scores of >5 (equating to ‘problem drinker’ or dependence). Of those reporting the number of units consumed (*n* = 3 studies), the range was 26.6 to 56.0 units per day. Three studies reported the number of heavy drinking years (13.1–17.0 years [24, 27, 28]), three discussed the history of alcohol‐related hospital presentation [27] or withdrawal seizures and delirium tremens [22, 28]. Three studies reported comorbid diagnosis of the patients included in the study, all of which may influence length of stay (LOS), response to interventions and ultimately the cost of the intervention. For example, incidence of renal and hepatic dysfunction [21] and rates of a history of depression [25, 26].

### 3.3 The intervention

Four studies reported patient and costing outcomes following the implementation of complex interventions for AWS management consisting of the provision of medications for symptom control AND any one of the following: counselling or psychological therapies OR access to Alcoholics Anonymous meetings OR thiamine provision OR assisted accommodation OR skill development training OR biochemical assessment of blood markers for alcohol dependence [24, 26, 27, 28]. One study described outcomes following a brief psychological intervention [23]. Three studies report outcomes following a pharmacological intervention for alcohol withdrawal, including the implementation of an inpatient chlordiazepoxide prescribing guideline [21, 22] and a fixed versus symptom‐triggered benzodiazepine (lorazepam or diazepam) prescribing design [25].

### 3.4 Alcohol‐related outcomes

Treatment outcome reporting was variable across the studies included (Table 2).

**TABLE 2:** Treatment‐related outcome domains.

Reference (intervention)	Alcohol consumption	Abstinence	Complications	Treatment completion	Intervention requirements, for example, LOS, engagement in intervention, medication requirements
Alwyn 2004 (community; psychological)	↓	↑			
Hayashida 1989 (inpatient vs. outpatient; complex)		↑ 1 month ↔ 6 months	↔	↑	↑ number of therapy sessions attended
Hoey 1994 (inpatient; pharmacological guideline)					↓ ICU stay
Soravia 2017 (residential; pharmacological guideline)					↓ acute and total LOS ↓ medication requirements
Nalpas 2003 (complex)		↔			
Klijnsma 1995 (outpatient; complex)	↔	↔			
Longabough 1983 (inpatient AW management and inpatient follow‐up vs. outpatient follow‐up; complex)		↑	↓		↔ duration of detox visit
Murdoch 2014 (inpatient; pharmacological guideline)			↓		↓ LOS ↓ medication prescribing

*Note*: Complex = complex interventions for AWS management consisting of provision of medications for symptom control AND any one of the following: counselling or psychological therapies OR access to Alcoholics Anonymous meetings OR thiamine provision OR assisted accommodation OR skill development training OR biochemical assessment of blood markers for alcohol dependence; Abbreviations: AW, alcohol withdrawal; LOS, length of stay.

Two studies reported negative findings following the implementation of their intervention. (i) When directly compared, treatment duration was significantly longer in those receiving outpatient detoxification compared with those receiving inpatient care [24]; and (ii) an increase in pharmacological burden (number of administrations required, number of patients requiring, or cumulative dose of medication) was also observed following the implementation of a chlordiazepoxide prescribing guideline; however, this was subsequently associated with a reduction in intensive care length of stay (LOS) [21] among the sub‐population requiring intensive care input.

Three of the five studies reporting changes in LOS or duration of engagement requirements for treatment were those employing pharmacological interventions; all three demonstrated a reduction in treatment duration [21, 22, 25]. Soravia et al. [25] and Murdoch et al. [22] also report: (i) reductions in the proportion of patients requiring pharmacological interventions; (ii) a reduction in the number of adjunct medications required; and (iii) reductions in the amount of, or duration over which, medications were prescribed. Reductions in complication rates such as reporting of severe alcohol withdrawal signs including seizures and delirium tremens, and post‐discharge alcohol‐related hospital reattendances and arrests were observed across two studies [22, 28]. Five studies reported outcomes relating to follow‐up post‐withdrawal management. Three specifically document changes in alcohol consumption through self‐reported measures; five report abstinence. Two studies employing psychological or talking therapy interventions report improvements in abstinence, including significant improvements in the number of days until first drink and the number of abstinent days [23] and an increase in the proportion of patients remaining abstinent and the number of abstinent days [28].

### 3.5 Economic evaluation methods

Of the eight papers included in this study, none included full health economic analyses; four included cost measures as primary outcomes [24, 25, 26, 28], and only one paper was identified as an economic evaluation [28]. Although several papers reported on interventions that were cost saving, with comparable clinical outcomes to treatment as usual (TAU = either clinician‐directed prescribing as opposed to guideline‐based, fixed‐dose benzodiazepine prescribing compared with symptom triggered, outpatient management with PRN benzodiazepine prescribing compared with inpatient management and extended inpatient withdrawal management compared with partial inpatient management [22, 24, 25, 26, 28]) only one study included a cost‐effectiveness calculation as part of the analysis [26]. This was based on the costs associated with providing residential rehabilitation from the French national social security service tariffs, length of stay, investigational costs and medication costs.

All included studies considered costs from a healthcare perspective; all but one study [21] evaluated service costs primarily by measuring contact time (either inpatient stays or outpatient attendances) and calculating costs using daily (inpatient) or hourly (outpatient) cost data. The cost data used was either collected as part of the trial [24, 26, 28], an average value calculated for the trial centre [23, 26, 27], or was derived from national tariffs [21, 25, 26, 27], with some studies [23, 26, 27] using a mixture of local and national data sources. One study did not report the source of cost data used [22].

Three studies included more detailed information on costing than just a standard contact time cost. Hayashida et al. [24] discussed the inclusion of a range of costs including screening visits, hospital treatment, ward costs, and included direct and indirect costs in their analysis. As well as calculating average service costs, this study also calculated marginal costs per treatment of an additional patient, included a sensitivity analysis and considered a wider societal/patient perspective, through the inclusion of patient transport and patient opportunity costs (what a patient had to give up in order to take part in the service), with a breakdown to average costs per treatment pathway for both clinic and patients. Nalpas et al. [26] used the same average daily rates for the four evaluated centres (based on national tariffs for hospital ward and employee costs), but also reported centre‐specific costs for inpatient program and supplementary medical visits, systematic and supplementary investigations and drugs costs. The study also used the effectiveness of treatment (mean length of time before relapse) to calculate efficiency costs per centre (total program care costs divided by mean time in months without relapse) to give a cost‐effectiveness comparison between centres. Longabaugh et al. [28] separated the costs for residential and treatment program attendance, enabling a comparison of the extended residential stay versus an outpatient treatment approach. This study also included tracking of readmission outcomes and associated costs.

Hoey et al. [21] reported solely on changes in the drug acquisition costs pre‐ and post‐change in drug implementation guidelines. Drug‐specific costs were also included in one other study [26]. Two studies quantified change in drug usage without calculating drug costs as part of service costs [22, 25]. Hoey et al. [21] recorded a change to average intensive care unit (ICU) stays as part of the intervention, but inpatient costs were not calculated as part of their analysis.

One study reported on additional staff costs for the intervention [23], based on the average staff hourly rate, but did not include staff training costs for intervention implementation. Another study commented on staff training and additional time for symptom‐triggered AWS detoxification, but staff costs were not included as part of their analysis [25].

### 3.6 Economic outcomes

Four papers reported on interventions where service settings impacted service costs. Hayshida et al. [24] directly compared inpatient and outpatient detoxification services from a randomised prospective study, where both service arms included daily adjusted drug doses, thiamine and counselling. While limited in detail, the groups between treatment arms were comparable in terms of their documented history of alcohol consumption (outpatient = age at first drink 15.4, years heavy drinking 13; inpatient = age at first drink 14.5, years heavy drinking 14.1). Those enrolled in the inpatient arm had a greater severity of alcohol withdrawal symptoms in previous withdrawal episodes and a greater number of mean intoxication days in the previous month [24]. The average service cost of outpatient treatment was reported as over a 12‐fold reduction in costs compared to inpatients ($3319–3665:$175–388), based on the difference in the average duration of treatment (9.2:6.5 days), the associated levels of residential administrative costs, and patient opportunity costs. The sensitivity range reported included a lower range calculated on average costs for visits to the detoxification centre, with the higher sensitivity range also including charges explicitly for detoxification, indirect costs and patient transport and opportunity costs. The study also reported a difference in marginal costs between the two pathways ($756–3047:$190–414); the low estimate based on adding an additional patient to the workload of existing units and the high estimate calculated at the cost of an additional patient in an institution already in full use, therefore requiring a new unit for that purpose. The extension of the outpatient program is reported as 4‐ to 7‐fold less expensive than inpatient treatment. Although significantly more inpatients completed detoxification (95%:72%), the proportion of completed follow‐up evaluations did not differ significantly; there was no difference reported in abstinence rates at 6 months for the two treatment pathways.

Loughabaugh et al. investigated the cost‐effectiveness of providing therapy either as an extended inpatient or an outpatient service after an initial inpatient detox period using a randomised controlled trial design [28]. No significant differences between the alcohol consumption characteristics were observed between the groups; an Urn model randomisation technique was employed to balance marital status and employment history between the groups [28]. Although initial inpatient detox costs were comparable for both groups ($1035:$1033), total treatment costs were 1.6‐fold lower for the outpatient group ($4359:$2700), which was driven by residential costs for the extended inpatient service. No differences were reported in re‐hospitalisation or multiple readmission outcomes at 6 months from the two arms, although costs for readmission to the program were again 1.3‐fold lower for the outpatient group than for the inpatient group ($2116:$1689), due to residential costs for inpatients.

The heterogeneity of complex alcohol treatment interventions was reflected by Nalpas et al., who compared treatment costs and outcomes at four specialised alcohol treatment centres [26]. The study reported a range of mean acute costs of €1327, €1764, €1917, €1398 and total costs of €3031, €2455, €2337, €1832, for centres 1–4, respectively. Within the acute phase, despite all four centres reporting a wide range of costs for inpatient programs and supplementary medical visits (€81–259), systematic and supplementary investigations (€43–306), and drugs (€30–99), the main driver was the average cost of the inpatient residential stay (€673, €1525, €1261 and €1154, for centres 1–4, respectively), which was driven by the LOS (11, 28, 19 and 19 days). Follow‐up costs were again driven predominantly by length of residential stay, with only one centre (1) including an extended inpatient stay, reflected in residential costs of €1343 compared with the other three centres (€196, €119 and €142, respectively). Intervention effectiveness (the mean time in months without relapse) was highest (5.89 months) for centre 1 (the most expensive centre; mean cost €3031) compared with only centre 4 (3.77 months; the cheapest centre; mean cost €1832). Comparing efficiency (or cost‐effectiveness) of the four interventions (calculated as total program care costs divided by effectiveness or mean time in months without relapse), centres 1, 3 and 4 are comparable (at around €500 per month without relapse), whereas centre 2 is less cost‐effective (averaging €658 per month without relapse). Although inpatient stay was a direct driver of service costs, cost‐effectiveness was impacted by a combination of intervention elements, which requires further investigation.

Kijnsma et al. [27] also reported a six‐fold reduction in costs for out‐patient detoxification (£235:£1404), although inpatient costs were based on national tariffs rather than trial data. Good or improved outcomes were reported for the majority of participants (17/28) although there was no TAU comparison.

Three studies report changes to service costs due to the implementation of prescribing or symptom management guidelines. Implementation of benzodiazepine selection guidelines (lorazepam to patients 60 or over or those with hepatic dysfunction, and chlordiazepoxide for all other patents) led to a 20‐fold decrease in drug acquisition costs per person ($1008.72 ± 1554.45: $59.79 ± 122.92), and was associated with a significant reduction in ICU days (4.1 days:1.1 days *p* < 0.0001) with no adverse effects attributable to changes in prescribing [21].

Additionally, A symptom‐triggered detoxification guideline led to a two‐fold reduction in healthcare costs per patient in the acute phase (CHF3,710.5 ± 1621.7:CHF1,797.8 ± 1137.4) and 1.4‐fold cost reduction for the entire inpatient stay (CHF12,509.8 ± 4048.1:CHF 9059.7 ± 3660.8) when compared with a fixed‐dose regimen [25]. This was secondary to a reduction in the acute detoxification phase duration (136(±59)h to 66(±42)h) and total LOS from 19.1 ± 6.2 to 13.8 ± 5.6 days. Although drug usage was not included in service costing, lorazepam administration was reported as decreasing from 14.8 mg in the TAU group to 5.1 mg (a 66% reduction) in the intervention group. There were no between‐group differences in complications (including seizures, hallucinations and delirium tremens) during detoxification or differences in levels of premature discontinuation.

Murdoch and Marsden [22] also reported that the implementation of a symptom‐triggered AWS protocol led to a 60% reduction in the duration of inpatient stays (6.4 days TAU [non‐symptom‐triggered detoxification protocol] to 2.5 days in AWS group), leading to a 2.6‐fold reduction of service costs (£1908:£722), based on inpatient stay day data only. A 66% reduction of chlordiazepoxide was also reported (563.3 to 167.2 mg), but drug usage was not included in service costs. A reduction of severe alcohol withdrawal signs (seizures and delirium tremens) was also reported.

Alwyn et al. [23] reported on the addition of a psychological community‐based intervention (motivational interviewing, coping skills training and social support) to an existing detoxification program; demonstrating a reduction in the time to first drink following treatment (average 114 days to 52 days, *p* = 0.011) and an increased percentage of patients remaining abstinent, or drinking 3 units a day or less at 12 months (7.5% to 39.5%). As the intervention was in addition to TAU, there was an increased service cost of £175 per participant (calculated on additional time of a community practice nurse, based on standard tariff rates). The significant improvements in outcomes suggest that this intervention may be cost‐effective, but this can not be determined, as savings costs due to improved outcomes were not included in the evaluation.

### 3.7 Study quality

The quality of included studies was evaluated using Consolidated Health Economic Evaluation Reporting Standards (CHEERS [20]). Due to most included studies being cost evaluations, rather than full economic analyses, many of the key CHEERS criteria were not met. Notably, questions surrounding study perspective, discounting and opportunity costs, and incremental analysis were only included in Hayashida et al. [24], with time horizons not included in any studies. For this reason, the quality of the studies from a health economics perspective is generally considered poor, seven or the included studies met less than 50% of the CHEERS guidelines (see Table 3), with only Hayashida et al. [24] considered of medium quality, as it met over 60% of the guidelines.

**TABLE 3:** CHEERS reporting guidance checklist for Health Economic Evaluations.

Citation	CHEERS item number																											Number of items missing	Percent of items missing
	1	2	3	4	5	6	7	8	9	10	11a	11b	12	13a	13b	14	15	16	17	18	19	20a	20b	21	22	23	24		
J Alwyn 2004		✔	✔	✔	✔		✔			✔		‐	‐		‐		‐	‐		‐			‐		✔			13	59
M Hayashida 1989	✔	✔	✔	✔	✔	✔	✔			✔	✔	‐	‐	✔	‐		‐	‐	✔		✔	✔	‐		✔			8	36
L Hoey 1994		✔	✔	✔			✔					‐	‐		‐		‐	‐			✔		‐		✔			15	68
L Soravia 2017	✔	✔	✔	✔	✔		✔			✔		‐	‐		‐		‐	‐			✔		‐		✔		✔	11	50
B Nalpas 2003	✔	✔	✔	✔	✔		✔				‐		‐		‐		‐	‐		✔	✔		‐		✔			12	50
M Klijnsma 1995			✔		✔		✔					‐	‐		‐		‐	‐					‐		✔			17	77
R Longabaugh 1983	✔		✔	✔	✔		✔			✔		‐	‐	✔	‐		‐	‐	✔				‐	✔	✔			11	50
J Murdoch 2014		✔	✔		✔		✔			✔		‐	‐		‐		‐	‐					‐		✔		✔	14	64
Number with missing items	4	2	0	2	1	7	0	8	8	3	6	1	‐	6	‐	8	‐	‐	6	6	4	7	‐	7	0	8	6		
Percent of items missing	50	25	0	25	13	88	0	100	100	38	75	13	‐	75	‐	100	‐	‐	75	75	50	88	‐	88	0	100	75		

*Note*: The checklist follows the guidelines set out in Husereau et al. (2022) [18]. =, item not identified (missing when expected); ✔ = item reported; − = not applicable; Colour Code: Blue = title and abstract, Yellow = introduction, Purple = methods, Green = results, Orange = discussion, Red = other (sources of funding and conflicts).

## 4 DISCUSSION

Following a systematic review of the existing scientific literature, eight studies reporting costing evaluations relating to AWS interventions and treatment outcomes were identified. These studies highlighted three main findings.

Firstly, the transfer of all or part of the treatment intervention to an outpatient facility was linked to a 1.6‐ to 12‐fold decrease in service costs. This was due to a reduction in the length of time spent in inpatient care, and the associated cost of inpatient residential stay and patient opportunity costs. The studies did not report any significant difference in intervention effectiveness compared to TAU (inpatient‐only treatment), indicating that the move to outpatient treatment may be cost‐effective. Some interventions leading to a reduction of inpatient duration, and therefore residential cost, were shown to be as clinically effective as standard practice in various treatment settings [24, 27]. For example, Hayashida et al. [24] directly contrast outcomes from inpatient and outpatient treatment pathways demonstrating comparable levels of clinical effectiveness in terms of abstinence rates while also achieving a 12‐fold reduction in costs. Specifically, outpatient care is associated with a reduction in patient‐incurred opportunity costs and an increase in the proportion of service costs attributable to marginal costs, for example, healthcare professionals' time due to service user home visits, as opposed to staff who are hospital‐based. However, these nuances were not accounted for in Klijnsma et al. [27] again underestimating the financial impact to both the service provider and the patient. We note that details relating to alcohol use and withdrawal management history are limited throughout. However, while Hayashida reports a comparable alcohol use history in terms of the number of heavy drinking years and age of initiation of heavy alcohol consumption, withdrawal histories were not comparable between treatment settings. Those in the inpatient setting experienced worse previous AWS [24]. A history of previous severe withdrawal symptoms is associated with more complex subsequent episodes [13, 29, 30]. As such, this may have impacted LOS or incurred costs of inpatient care and therefore overall costs attributed to this treatment arm.

Secondly, the implementation of structured (and symptom‐based [25]) benzodiazepine‐based prescribing guidelines for AWS interventions in acute inpatient settings was associated with a reported reduction of between 2‐ and 20‐fold in service costs compared with no guideline (or a fixed‐dose prescribing regimens [25]). Again, the decrease in cost was due to a decrease in associated residential costs. The studies largely reported no significant difference in AWS‐related complications; a single study demonstrated an improvement. The reduction of costs with comparable or improved outcomes suggests that these interventions may be more cost‐effective when compared with alternative approaches to AWS management. Hoey et al. [21] reported that structured prescribing guideline implementation led to a reduction of drug acquisition costs by transferring patients from intravenous lorazepam infusion to intravenous or oral chlordiazepoxide, which was cheaper in this treatment setting. However, the proportion of those receiving intravenous chlordiazepoxide versus oral formulations is not described. With these prescribing changes, there is also likely to be a cost reduction due to practice‐based environmental constraints, as intravenous lorazepam administration is often restricted to more expensive ICU or high‐dependency wards where continuous monitoring and improved staffing ratios are available, whereas oral chlordiazepoxide can be administered outside of these environments. This may be a key driver for the reduction of mean ICU days reported in this study, although length of ICU stay was not incorporated into the study costings. Dasta et al. [31] also report that ICU cost per day ranged between 12,931 and 42,570 USD in 2002 depending on ventilation requirements. As such, the implementation of the structured prescribing guideline likely had significant cost implications for the service. Both of the system‐triggered management guidelines also report a 66% drug dosage reduction required, which also needs to be considered when calculating the cost‐effectiveness of these interventions.

Finally, the addition of psychological intervention to TAU was associated with increased periods of abstinence following withdrawal management and a reduction in alcohol consumption, indicating that the addition of a psychological support program at a relatively low additional cost may be an effective way of improving the cost‐effectiveness of existing services.

The overall findings from these studies demonstrate that the main driver of more cost‐effective programs is the ability to reduce the number of inpatient days. However, the absence of an in‐depth economic evaluation in these studies limits their impact. In addition, taking a wider economic perspective, including patient opportunity costs, as was considered in Hayashida et al. [24], may more accurately reflect real‐world cost savings for these types of interventions.

### 4.1 Strengths and weaknesses

To our knowledge, this is the first review to demonstrate that intervention location and structured AWS prescribing guidelines can be used to reduce the cost of the AWS management program. The cost reductions of both approaches are driven by a decrease in inpatient time (and associated residential costs), without impacting on AWS symptoms or long‐term outcomes.

We do, however, acknowledge weaknesses to this study. A full health economic evaluation was infrequently employed. As such, the wider context of the costs associated with delivering the intervention in question, beyond those attributed to the service provider, was not determined in the majority of studies. While the transfer of treatment to outpatient facilities was associated with a reduction in costs, limited consideration around patient demographics and alcohol use history was taken as part of any cost calculations, limiting generalisability and conclusions surrounding service cost efficiency. For example, the transfer of care to outpatient facilities may not be clinically appropriate for those considered high‐risk of alcohol withdrawal‐related adverse events, such as seizures or delirium tremens. Furthermore, patients considered highly physically dependent on alcohol would be more likely to be offered inpatient management and experience lengthier hospital stays, both of which would be associated with higher treatment costs and impact service cost efficiency. Very few of the studies included report outcomes from recent retrospective or prospective data sets, employing robust and evolved health economic evaluation methods. While not only limiting translation to modern health services and treatment settings, our understanding of the characteristics of those presenting to alcohol services since the publication of some of the included studies has developed significantly. As such, the applicability to the population of those experiencing AWS may also be limited. The papers included have significant heterogeneity among study methodology, patient population, intervention type, and setting. It is not possible to understand which elements of complex interventions are more strongly linked to any reported improved outcomes. The overall quality of papers included, as defined by the CHEERS health economic evaluation checklist, is poor. Most papers include only limited cost measurements, and only one paper actually includes a cost‐effectiveness evaluation. For this reason, although current findings show the potential to improve the cost‐effectiveness of alcohol withdrawal programs, further evaluations, with embedded health economics, are required to investigate potential effectiveness and cost‐effectiveness improvements to these types of intervention. As grey literature was excluded from the study, we acknowledge that this may have reduced identification of health economic evaluations of interventions that were not performed in academic settings; for example, end‐of‐year private rehabilitation centre financial reports.

### 4.2 Recommendations

Across the studies included, various themes have emerged highlighting the relative importance of finding a balance between intervention variables to achieve a clinically impactful outcome and cost‐effective treatments. Based on the heterogeneity of study characteristics and context, it was not possible to determine overall cost and clinical effectiveness; for example, cost per successful treatment outcome of a single alcohol‐dependent individual.

**FIGURE 2:**
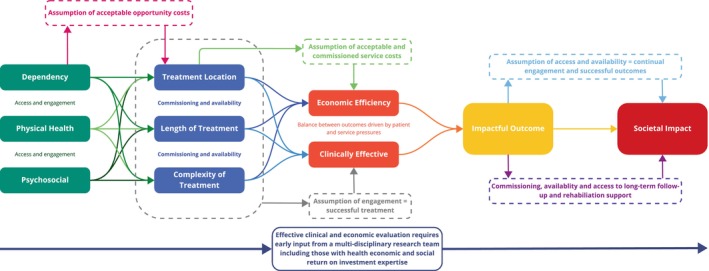
Logic model of factors that impact AWS treatment. The relationship between various patient and service factors is explored. The central determinants of overall societal impact and treatment success are patient factors (green). These, alongside assumptions of acceptable opportunity costs to the patient and local commissioning and service availability, will influence engagement and treatment setting, complexity and duration (blue). Here we demonstrate the treatment setting, pharmacological prescribing protocols and the addition of psychological interventions, may be drivers of economic efficiency; while also maintaining clinical effectiveness (orange). Assumptions surrounding the economic acceptability of commissioned services and that engagement with services leads to successful treatments, influence the linearity of this relationship and the likelihood of an impactful outcome (yellow). Furthermore, the ability of successful alcohol detoxification treatment outcomes to translate to long‐term sobriety and social impact (red; through reintegration and independence, for example), assumes continual access and engagement with services, and commissioning and availability of these services long‐term.

Patient factors such as duration and severity of alcohol dependence, additional physical health needs and psychosocial situation are all primary mediators in both cost and clinical effectiveness of interventions for AWS (Figure 2). Individuals with more complex alcohol use histories and AWS‐related presentations are at increased risk of developing severe symptoms of AWS in subsequent episodes [32, 33, 34]. Worse outcomes are also observed in those presenting with AWS alongside another co‐morbid condition, for example, non‐elective trauma [35]. Furthermore, psychosocial factors such as unemployment [36] and a dual diagnosis of AWS alongside other psychiatric conditions may be associated with increased severity of AWS presentations (e.g., co‐occurring AWS and anxiety disorder diagnoses [37]). As such, the suitability of AWS intervention and intervention location will ultimately be determined by patient characteristics, and medical and psychosocial risk factors. With increasing levels of dependence severity, physical health, and psychosocial complexity associated with an increasing need for medical AWS oversight, likelihood for ICU involvement, longer LOS, and decreased likelihood of long‐term abstinence and psychosocial reintegration, all of which we demonstrate significantly influence cost. In addition to patient and service‐specific factors, various assumptions are inherently made relating to patient engagement and treatment outcomes, which also impact clinical and cost‐effectiveness. For example, the assumption that treatment service commissioning and availability will lead to access and engagement when readiness to change behaviours may also strongly predict engagement in treatment and changes in alcohol use outcomes [38, 39]. Or that engagement in alcohol services leads to long‐term sobriety and psychosocial rehabilitation [5, 32, 40, 41]. To evaluate clinical and economic effectiveness more broadly, studies taking a holistic view of service development, patient recovery trajectory and onward rehabilitation are needed. Economic evaluations that consider a wider societal perspective or include measures of social value, such as social return on investment methodologies, could facilitate this [42, 43].

A model of overall clinical‐, societal‐ and cost‐effectiveness requires in‐depth knowledge of patient characteristics, intervention parameters, appropriate clinical and economic assessment metrics, and long‐term follow‐up outcomes. Our findings build upon this model, suggesting that the following assumptions are also made when determining the impact of AWS services on patients and health services: (i) that opportunity costs are equitable and acceptable; (ii) that commissioned services are acceptable to patients; (iii) that engagement in those services leads to successful outcomes; and (iv) service commissioning for AWS services will lead to continual engagement and successful outcomes. Based on these findings, further work is needed to develop effective and translatable cost‐effectiveness models for AWS and alcohol‐related care pathways to justify commissioning and stratify treatment decisions. Steps towards this would include:

1. Further exploration of risk‐factors predictive of successful community, inpatient or residential alcohol use disorder treatment programs. Thus, optimising cost‐effectiveness of current pathways through improved ‘success’ rates.
2. The active engagement of individuals and groups with expertise in economic evaluation and determining social return on investment in service and intervention evaluation studies in research surrounding AWS management and treatment development will improve the impact made possible from available, retrospective data sets, which we demonstrate is currently limited without such expertise. Furthermore, early recruitment of experts with the skill sets will improve economic cost data collection and recording, providing a richer, more holistic assessment of the clinical and cost‐effectiveness of interventions overall.

## AUTHOR CONTRIBUTIONS

The study concept, study design and evaluation methodology were conceived and developed by Rhiannon Tudor Edwards, Darren Quelch, Rachel Granger, and Huw Lloyd‐Williams. Darren Quelch, Rachel Granger and Huw Lloyd‐Williams conducted literature screening. Darren Quelch and Rachel Granger completed the data extraction and the first draft of the manuscript. All authors provided edits and critiqued the manuscript and all authors have read and agreed to the published version of the manuscript. Each author certifies that their contributions to this work meet the standards of the International Committee of Medical Journal Editors.

## CONFLICT OF INTEREST STATEMENT

The authors received no specific funding for this work. Darren Quelch has no COI to declare; Rachel Granger has no COI to declare; Huw Lloyd Williams has no COI to declare; Rhiannon Tudor Edwards has no COI to declare.

## Supporting information


**Table S1.** Inclusion and exclusion criteria.
**Table S2.** Search terms.

## Data Availability

Data surrounding systematic review methodology and outcomes will be made available by request to the author.
