# Supplementary material for: A systematic review of the economic evidence surrounding the management of alcohol withdrawal
Source: Drug Alcohol Rev. 2025 Apr 14;44(4):990–1009. doi: 10.1111/dar.14053 (PMC12117296; doi:10.1111/dar.14053)
Supplement: Supplementary file 1 — Table S1. Inclusion and exclusion criteria. Table S2. Search terms. [file DAR-44-990-s001.docx]

**Supporting Information**

**Table S1. Inclusion and exclusion criteria**

| STUDY TYPE | |
| --- | --- |
| Inclusion | **Exclusion** |
| Clinical audit | Self-reports |
| Service evaluation | Mainstream media reports |
| National audit | Blog entries |
| Intervention trial | Social media |
| Economic evaluation | Systematic review articles (+/-meta-analysis) |
| Cost-effectiveness study | Narrative review articles |
| Social return on investment study |  |
| SETTING – Information Source / Research Setting | |
| Inclusion | **Exclusion** |
| Inpatient | Mainstream media news reports |
| Outpatient | Patient recollection |
| Community | Individual reports – Case reports |
| General practice | Pre-clinical |
| Residential rehabilitation | Animal studies – *in vivo* and *ex vivo* |
| Charity services | *In vitro* studies |
| Acute medical | Perinatal services |
| Addiction psychiatry | Paediatric services |
| Addiction services |  |
| Third sector |  |
| Alcohol care teams |  |
| Assertive outreach teams |  |
| Hepatology services |  |
| Community addiction services |  |
| Hospital reports |  |
| PATIENT – Demographics | |
| Inclusion | **Exclusion** |
| Male | ≤ 18 years of age |
| Female | Perinatal |
| ≥18 years of age |  |
| PATIENT – Diagnosis Characteristics | |
| Inclusion | **Exclusion** |
| Alcohol withdrawal | Social drinkers |
| Delirium tremens | Alcohol intoxication without dependence |
| Alcohol detoxification | Hazardous drinking |
| Alcohol withdrawal syndrome | Recreational drinking |
| Poly-substance addiction with alcohol withdrawal syndrome |  |
| Admissions with a history of harmful alcohol consumption |  |
| INTERVENTION – Substance | |
| Inclusion | **Exclusion** |
| Detoxification – any setting | Alcohol consumption reduction service or intervention |
| Fixed dose benzodiazepine | Abstinence service |
| Symptom triggered benzodiazepine | Sobriety service |
| Symptom monitoring |  |
| Medically assisted withdrawal |  |
| Psychological intervention |  |
| INTERVENTION – Comparator | |
| Inclusion | **Exclusion** |
| Detoxification – any setting if multiple service settings found during literature searching | Alcohol consumption reduction service or intervention |
|  | Abstinence service |
|  | Sobriety service |
|  | Rehabilitation following withdrawal management |
| OUTCOMES – Financial | |
| Inclusion | **Exclusion** |
| Costs in monetary value |  |
| Costs in patient or healthcare professional time |  |
| Costs in hospital length of stay |  |
| Quality adjusted life years |  |
| Cost-benefit ratio |  |
| OUTCOMES – Societal | |
| Inclusion | **Exclusion** |
| Access to treatment |  |
| Population served |  |
| Savings to other services |  |
| Social return on investment |  |

**Table S2. Search terms**

| Search terms | | | | |
| --- | --- | --- | --- | --- |
|  | **Key terms** | **Indexed terms** | | |
|  |  | **MEDLINE (PubMed)**  **https://meshb.nlm.nih.gov/search** | **PsychInfo (https://www-proquest-com.ergo.southwales.ac.uk/psycinfo/advanced?accountid=15324)** | **Embase (Emtree terms https://www-embase-com.ergo.southwales.ac.uk/emtree?term=social+return&results=true)** |
| Population | ‘alcohol-induced delirium’  Detox*  ‘alcohol withdrawal’  Withdrawal  ‘Delirium Tremens’  ‘Alcohol detox’  ‘alcohol detoxification’  Wernicke’s encephalopathy  ‘withdrawal seizure’  ICD 6C40.4 Alcohol withdrawal  ICD 6C40.5 Alcohol-induced delirium | Alcohol Withdrawal Delirium [MeSH Terms]  Alcohol-Induced Disorders [MeSH Terms]  Wernicke Encephalopathy [MeSH Terms]  Alcohol-Induced Disorders, Nervous System[MeSH Terms]  Alcohol Withdrawal Seizures[MeSH Terms] | MAINSUBJECT.EXACT("Detoxification") MAINSUBJECT.EXACT("Alcohol Withdrawal") MAINSUBJECT.EXACT("Wernicke Encephalopathy")MAINSUBJECT.EXACT("Alcohol Withdrawal Syndrome") | 'alcoholic delirium'/exp  'Wernicke  'Wernicke encephalopathy'/exp  'withdrawal syndrome'/exp 'alcohol withdrawal syndrome'/exp 'drug detoxification'/exp 'delirium tremens'/exp |
| Intervention | ‘Community withdrawal’  Community detox*  Residential detox*  ‘Residential alcohol withdrawal’  ‘Medically assisted withdrawal’  ‘Inpatient withdrawal’  Inpatient detox*  Elective detox*  ‘Symptom triggered’  ‘Fixed dose regimen’  ACT  Community alcohol  Assertive outreach  Alcohol liaison  Inpatient addiction  ‘Inpatient alcohol service’  ‘Alcohol Care Team’  Psychological interventions | Residential Treatment[MeSH Terms]  Assertive community treatment[MeSH Terms]  Community mental health services[MeSH Terms]  Community psychiatry[MeSH Terms] | MAINSUBJECT.EXACT("Assertive Community Treatment") MAINSUBJECT.EXACT("Residential Care Institutions") MAINSUBJECT.EXACT("Community Mental Health") MAINSUBJECT.EXACT("Addiction Medicine") MAINSUBJECT.EXACT("Community Psychiatry") MAINSUBJECT.EXACT("Community Psychology") MAINSUBJECT.EXACT("Medication-Assisted Treatment") | 'treatment withdrawal'/exp  'community mental health service'/exp 'residential care'/exp 'assertive community treatment'/exp |
| Comparators | Standard care  Symptom monitoring |  |  |  |
| Outcome | Cost*  Pounds  Dollars  Euro  Economic evaluation  ‘Financial evaluation’  Finance*  Economic*  ‘Cost benefit ratio’  Length of stay  Healthcare professional hours  Quality adjusted life years  QALY  Cost-benefit ratio  ‘Social return on investment’  SROI  ‘Service access’ | Models, economic[MeSH Terms]  Economics, hospital[MeSH Terms]  Economics, medical[MeSH Terms]  Health care economics[MeSH Terms]  Quality-Adjusted Life Years[MeSH Terms]  Health care costs[MeSH Terms]  Cost-benefit analysis[MeSH Terms] | MAINSUBJECT.EXACT("Costs and Cost Analysis") MAINSUBJECT.EXACT("Health Care Economics") | 'economic evaluation'/exp 'quality adjusted life year'/exp |
